# Supplementary material for: Association of SARS-CoV-2 vaccination status with risk of influenza-like illness and loss of workdays in healthcare workers
Source: Commun Med (Lond). 2025 Aug 9;5:347. doi: 10.1038/s43856-025-01046-8 (PMC12335509; doi:10.1038/s43856-025-01046-8)
Supplement: Supplementary file 2 — Supplementary Information [file 43856_2025_1046_MOESM2_ESM.pdf]

SUPPLEMENTARY MATERIAL

Supplementary figures

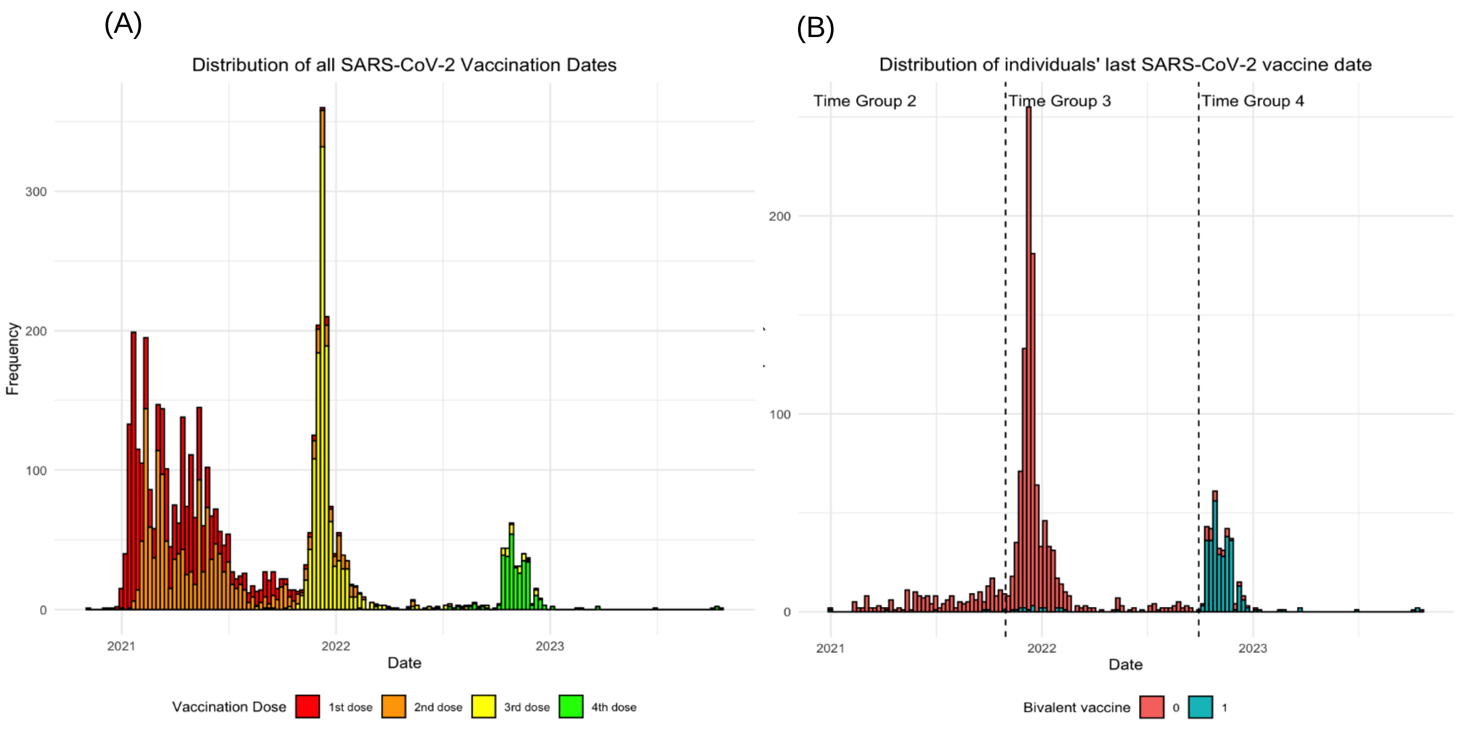

Figure S1. Distribution of SARS-CoV-2 vaccination dates over time (A). Distribution of last vaccine date (B). For the variable "time of last vaccination", dates of the official recommendations by the Swiss Federal Office of Public Health (FOPH) were used as cut-offs: primary vaccination course being recommended for all adults since January 2021 (2 doses or 1 dose in case of previous infection); booster vaccination recommended for all adults recommended on November 26th, 2021; additional booster vaccination recommended for risk population (including HCW) on October 10th, 2022.

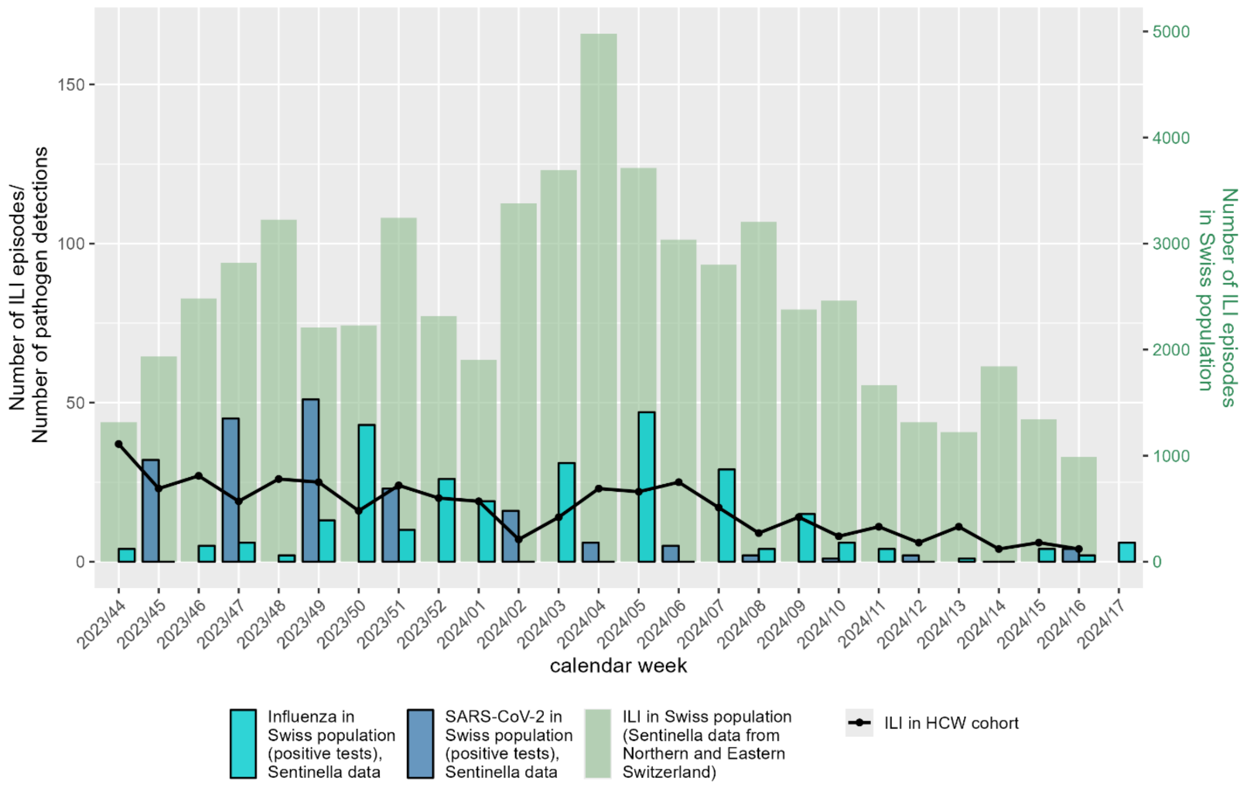

Figure S2. ILI episodes in the HCW cohort (black line) as compared to the local epidemiology in Northern and Eastern Switzerland according to Sentinella surveillance system (data available from: https://www.idd.bag.admin.ch/diseases/influenza/overview).

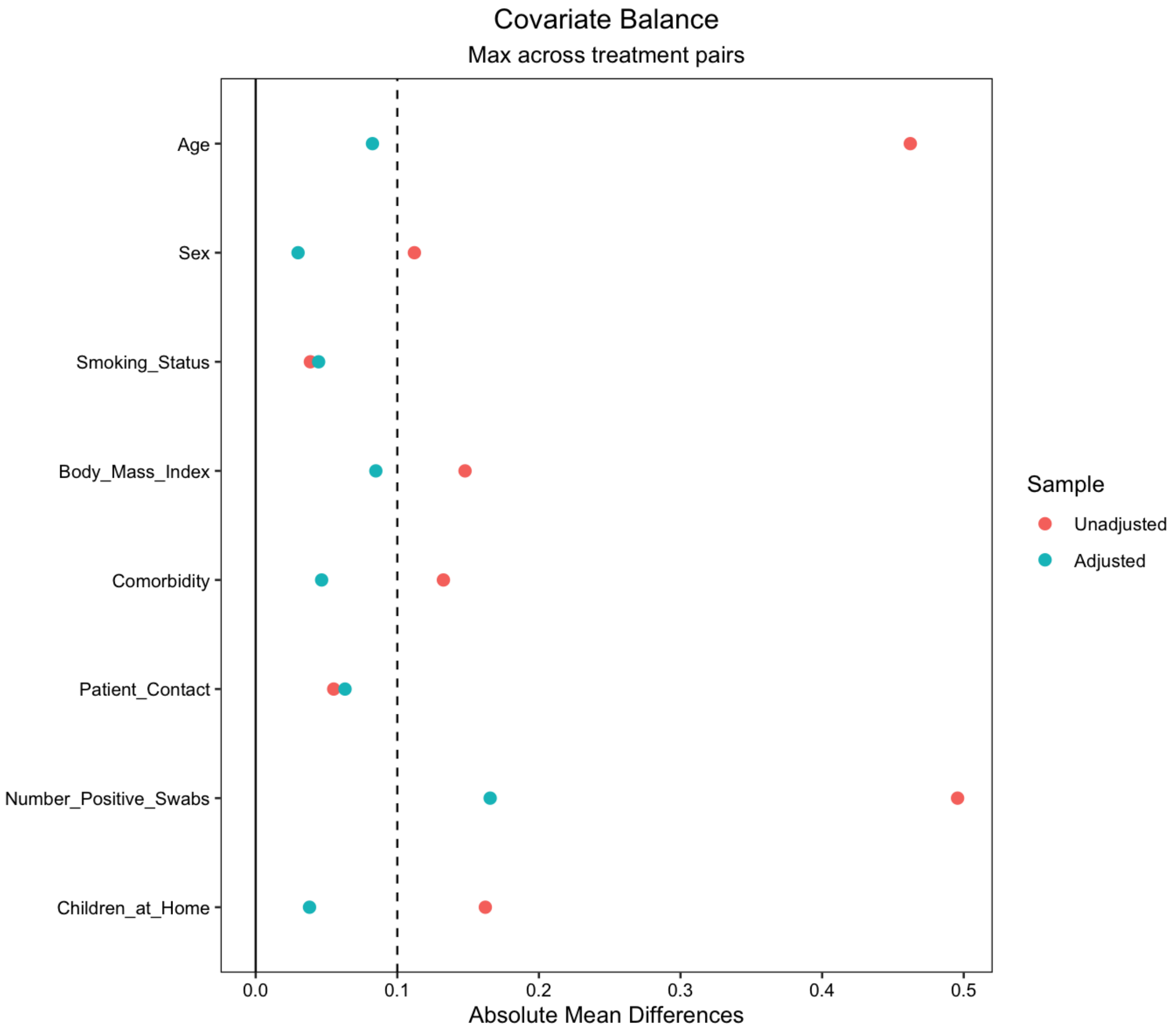

Figure S3. Balance plot to visualize covariate balance before and after weighting (unadjusted = unweighted, adjusted=weighted)

Supplementary tables

Table S1. Symptoms collected in weekly questionnaires

**General symptoms**	**Respiratory symptoms**
Feverishness	Cough
Body temperature ≥38° C	Runny or congested nose
Chills or shivering	Sneezing
Headache	Sore throat
Myalgia or aching limbs	Dyspnea
Palpitations or intensified heartbeat	Pain in chest, thorax and/or sternum
Fatigue	
Arthralgia	
**Other symptoms**	**Gastrointestinal symptoms**
Loss of smell and/or taste	Anorexia/Loss of appetite
Reddening or itching of eye(s)	Nausea and/or vomiting
Rash	Diarrhea
Other (text field to indicate which ones)	Stomach pain

Table S2. Co-variable definitions, levels, and time points when variables were obtained. All variables collected in October 2023, influenza vaccination was considered also during follow-up period.

Variable name (unit)	Definition	Levels
Age (years)	Age reached in 2023	cont.
Body mass index (kg/m2)	Body mass index at time of study entry	cont.
Sex	Biological attribute	Female; Male (ref[a])
Comorbidities	Presence of any of the following: arterial hypertension, diabetes, cancer, pulmonary disease, immune deficiency, cardiovascular disease	Yes; No (ref)
Active smoking	Self-reported active smoker	Yes; No (ref)
Patient contact	Working in patient rooms or facing patients as administrative worker	Yes; No (ref)
Home with children	Living with children < 13 years in the same household	Yes; No (ref)
Number of positive swabs	Number of positive SARS-CoV-2 swabs (at least 4 weeks apart) since the beginning of the pandemic until October 2023	continuous
Influenza vaccine	Receipt of seasonal influenza vaccination (2023/2024)	Yes; No (ref)
SARS-CoV-2 vaccinations	Number of SARS-CoV-2 vaccinations	0; 1 or 2; 3; 4 (categ.)
Time of last vaccine	Time of last SARS-CoV-2 vaccine, grouped. 1, no vaccination; 2, last vaccine before Nov 2021; 3, last vaccine Nov 20212 – Sep 2022; 4, last vaccine after Sep 2022 (see Figure S1)	1, 2, 3, 4 (cont.)

ref, reference; cont, continuous; categ, categorical

Table S3. Number of Influenza-like Illness episodes and workdays lost by SARS-CoV-2 vaccination status (raw data for Figure 2).

	**Unvaccinated N=175**	**1 or 2 doses N=364**	**3 doses N=895**	**4 doses N=311**
Number of ILI episodes	*n*	*n*	*n*	*n*
0	118	205	494	180
1	38	115	254	74
2	11	29	105	33
3	5	6	33	18
4	2	7	4	4
5	1	2	4	2
6	0	0	1	0
Number of days absent	*n*	*n*	*n*	*n*
0	96	197	439	160
1	21	36	108	35
2	16	38	93	43
3	9	20	53	19
4	7	15	47	11
5	6	13	29	7
6	6	9	26	7
7	8	7	23	6
8	1	7	14	5

Table S4. Sensitivity analysis with a more lenient ILI definition

**Characteristics**	**aIRR**[1]	**95% CI**[1]	**p-value**
Number of Vaccines			
0	—	—	
1	1.31	1.01, 1.72	0.046
3	1.52	1.19, 1.96	<0.001
4	1.67	1.26, 2.23	<0.001
Age	0.98	0.98, 0.99	<0.001
Sex	1.07	0.90, 1.26	0.4
Body Mass Index (kg/m2)	1.01	1.0, 1.02	0.3
Any Comorbidity	1.17	0.99, 1.37	0.062
Active Smoking	1.08	0.90, 1.30	0.4
Number of Positive SARS-CoV-2 Swabs	1.18	1.08, 1.28	<0.001
Patient Contact	1.03	0.89, 1.19	0.7
Home with Children	0.95	0.81, 1.11	0.5
Influenza vaccination season 2023/2024	0.87	0.74, 1.02	0.077

[1]aIRR = adjusted Incidence Rate Ratio, CI = Confidence Interval

Table S5. Sensitivity analysis with a more restrictive ILI definition

**Characteristics**	**aIRR**[1]	**95% CI**[1]	**p-value**
Number of Vaccines			
0	—	—	
1	1.25	0.94, 1.67	0.12
3	1.50	1.16, 1.96	0.002
4	1.55	1.15, 2.11	0.004
Age	0.98	0.97, 0.99	<0.001
Sex	1.04	0.87, 1.25	0.6
Body Mass Index (kg/m2)	1.01	0.99, 1.02	0.3
Any Comorbidity	1.22	1.02, 1.45	0.024
Active Smoking	1.12	0.92, 1.36	0.3
Number of Positive SARS-CoV-2 Swabs	1.20	1.09, 1.31	<0.001
Patient Contact	1.06	0.91, 1.24	0.5
Home with Children	0.98	0.83, 1.15	0.8
Influenza vaccination season 2023/2024	0.85	0.71, 1.01	0.062

[1]aIRR = adjusted Incidence Rate Ratio, CI = Confidence Interval

Table S6. Sensitivity analysis for evaluation of the effect of bivalent vaccine formulation avoiding multi-collinearity by grouping those with 3 or 4 doses of SARS-CoV-2 vaccination together.

	Model 1: Without time			Model 2: With time		
**Characteristics**	**aIRR[1]**	**95% CI[1]**	**p-value**	**aIRR[1]**	**95% CI[1]**	**p-value**
Time of last Vaccine				1.15	0.93, 1.42	0.19
Number of Vaccines						
0	—	—		—	—	
1 or 2	1.28	0.97, 1.69	0.08	1.05	0.69, 1.58	0.84
3 or 4	1.53	1.19, 1.98	0.001	1.15	0.69, 1.89	0.59
Receipt of ≥1 doses of bivalent vaccine	1.20	1.01, 1.43	0.04	1.06	0.82, 1.37	0.63
Age	0.98	0.98, 0.99	<0.001	0.98	0.97, 0.99	<0.001
Sex	1.11	0.93, 1.32	0.23	1.11	0.94, 1.32	0.22
Body Mass Index (kg/m2)	1.01	1.00, 1.02	0.15	1.01	1.00, 1.02	0.15
Any Comorbidity	1.19	1.00, 1.40	0.04	1.18	1.00, 1.40	0.05
Active Smoking	1.12	0.92, 1.34	0.25	1.11	0.92, 1.34	0.27
Number of Positive SARS-CoV-2 Swabs	1.21	1.11, 1.32	<0.001	1.21	1.11, 1.32	<0.001
Patient Contact	1.02	0.88, 1.18	0.82	1.02	0.88, 1.18	0.84
Home with Children	0.95	0.81, 1.11	0.54	0.96	0.70, 0.97	0.59
Influenza vaccination season 2023/2024	0.83	0.70, 0.97	0.02	0.82	0.70, 0.97	0.02

[1]aIRR = adjusted Incidence Rate Ratio, CI = Confidence Interval

Table S7. Subgroup analysis (model 1) for only influenza unvaccinated individuals.

Characteristics	aIRR[1]	95% CI[1]	p-value
Number of Vaccines			
0	—	—	
1	1.30	0.98, 1.73	0.069
3	1.54	1.19, 2.01	0.001
4	1.60	1.16, 2.22	0.005
Age	0.98	0.97, 0.99	<0.001
Sex	1.10	0.89, 1.35	0.4
Body Mass Index (kg/m2)	1.01	1.00, 1.03	0.085
Any Comorbidity	1.20	0.98, 1.45	0.070
Active Smoking	1.03	0.83, 1.26	0.8
Number of Positive SARS-CoV-2 Swabs	1.20	1.08, 1.33	<0.001
Patient Contact	1.03	0.87, 1.22	0.8
Home with Children	0.88	0.73, 1.06	0.2

[1]aIRR = adjusted Incidence Rate Ratio, CI = Confidence Interval
