# Supplementary material for: Association of SARS-CoV-2 vaccination status with risk of influenza-like illness and loss of workdays in healthcare workers
Source: Commun Med (Lond). 2025 Aug 9;5:347. doi: 10.1038/s43856-025-01046-8 (PMC12335509; doi:10.1038/s43856-025-01046-8)
Supplement: Supplementary file 3 — Reporting summary [file 43856_2025_1046_MOESM3_ESM.pdf]

nature portfolio

Corresponding author(s): Philipp Kohler

Last updated by author(s): June 26, 2025

Reporting Summary

Nature Portfolio wishes to improve the reproducibility of the work that we publish. This form provides structure for consistency and transparency in reporting. For further information on Nature Portfolio policies, see our Editorial Policies and the Editorial Policy Checklist.

Statistics

For all statistical analyses, confirm that the following items are present in the figure legend, table legend, main text, or Methods section.

n/a	Confirmed	
☐	☒	The exact sample size (*n*) for each experimental group/condition, given as a discrete number and unit of measurement
☐	☒	A statement on whether measurements were taken from distinct samples or whether the same sample was measured repeatedly
☐	☒	The statistical test(s) used AND whether they are one- or two-sided<br>*Only common tests should be described solely by name; describe more complex techniques in the Methods section.*
☐	☒	A description of all covariates tested
☐	☒	A description of any assumptions or corrections, such as tests of normality and adjustment for multiple comparisons
☐	☒	A full description of the statistical parameters including central tendency (e.g. means) or other basic estimates (e.g. regression coefficient) AND variation (e.g. standard deviation) or associated estimates of uncertainty (e.g. confidence intervals)
☐	☒	For null hypothesis testing, the test statistic (e.g. *F*, *t*, *r*) with confidence intervals, effect sizes, degrees of freedom and *P* value noted<br>*Give P values as exact values whenever suitable.*
☒	☐	For Bayesian analysis, information on the choice of priors and Markov chain Monte Carlo settings
☒	☐	For hierarchical and complex designs, identification of the appropriate level for tests and full reporting of outcomes
☐	☒	Estimates of effect sizes (e.g. Cohen's *d*, Pearson's *r*), indicating how they were calculated

Our web collection on statistics for biologists contains articles on many of the points above.

Software and code

Policy information about availability of computer code

Data collection	Data collection via online questionnaires, distributed and stored via RedCAP database version 14.0.42 provided by CTU Bern, Department of Clinical Research, University of Bern.
Data analysis	R version 4.4.0, R studio vesion 2024.04.2+764, packages 'tableone', 'nlme', 'MASS' and 'twang'

For manuscripts utilizing custom algorithms or software that are central to the research but not yet described in published literature, software must be made available to editors and reviewers. We strongly encourage code deposition in a community repository (e.g. GitHub). See the Nature Portfolio guidelines for submitting code & software for further information.

Data

Policy information about availability of data

All manuscripts must include a data availability statement. This statement should provide the following information, where applicable:

- Accession codes, unique identifiers, or web links for publicly available datasets
- A description of any restrictions on data availability
- For clinical datasets or third party data, please ensure that the statement adheres to our policy

The data is available from the corresponding author upon reasonable request.

Research involving human participants, their data, or biological material

Policy information about studies with human participants or human data. See also policy information about sex, gender (identity/presentation), and sexual orientation and race, ethnicity and racism.

Reporting on sex and gender	The research findings apply to both males and females. Participants self-identified their biological sex in the baseline questionnaire(s), with 1424/1745 (81.6%) of the cohort self-identifying as female. Sex was considered an important confounding factor, so it was included in the multivariable models. However, we did not conduct separate sex-based analyses, as the analysis adequately accounted for the influence of sex.
Reporting on race, ethnicity, or other socially relevant groupings	All participants self-reported their ethnicity in the baseline questionnaire at inclusion. Overall, 98.9% reported to be European, 0.7% reported Asian ethnicity, the remaining 0.4% identifying as either African, Hispanic or miscellaneous. Since this characteristic was so homogenous it was not considered as a confounding factor for the analysis and not included in the mutlivariable models.
Population characteristics	The study population consisted of 1745 healthcare workers (HCW), out of which 2113 completed in the baseline questionnaire in October 2023 with a median age of 46 years (range 17-70 years) and 81.6% being female. Additionally, 13.8% were active smokers, 19.8% had at least one comorbidity, the professional breakdown was 12.3% physicians, 46.0% nurses, and the remaining were therapists, administrative workers, and other roles. Direct patient contact was reported by 69.4% and 25.8% had received the seasonal influenza vaccine for the 2023/2024 season. The cohort included 175 (10.0%) participants unvaccinated for SARS-CoV-2, 364 (20.9%) with 1-2 vaccine doses, 895 (51.3%) with 3 doses and 311 (17.8%) with 4 doses of SARS-CoV-2 vaccines.
Recruitment	The study participants were recruited from a multicenter healthcare worker (HCW) cohort from nine healthcare networks in Northern and Eastern Switzerland which had started enrolment in August 2020. All hospital employees aged 16 years and older, with and without direct patient contact, were eligible for inclusion in the cohort upon providing informed consent. In October 2023, all new and ongoing participants in the cohort provided or updated their baseline data. This October 2023 cohort was then used as the study population, with certain exclusion criteria applied. The self-selection of participants into the study could have introduced bias, as those who choose to enroll may differ systematically from those who did not.
Ethics oversight	The study was approved by the Ethics Committee of Eastern Switzerland (#2020-00502).

Note that full information on the approval of the study protocol must also be provided in the manuscript.

Field-specific reporting

Please select the one below that is the best fit for your research. If you are not sure, read the appropriate sections before making your selection.

☒ Life sciences ☐ Behavioural & social sciences ☐ Ecological, evolutionary & environmental sciences

For a reference copy of the document with all sections, see nature.com/documents/nr-reporting-summary-flat.pdf

Life sciences study design

All studies must disclose on these points even when the disclosure is negative.

Sample size	The final sample size for this observational study consisted of 1745 participants, after applying the exclusion criteria described below. The initial cohort included 2113 participants who completed the baseline questionnaire in October 2023. The sample size was determined based on the participants in our cohort rather than a pre-specified power calculation, given the observational nature of the study.
Data exclusions	We excluded participants who provided less than 50% of follow-up questionnaires to ensure sufficient data for accurate outcome assessment. We also excluded individuals that reported having had more than four vaccinations, as five or more doses were only recommended for a highly specific population subset by the Swiss authorities. Additionally, participants receiving SARS-CoV-2 vaccination during the follow-up period were excluded in order to prevent confounding and bias in the causal relationship between vaccination status and outcomes.
Replication	Due to the lack of an independent cohort with similar baseline characteristics and outcome data, the reproducibility of the findings cannot be verified in this study.
Randomization	Not applicable in this study which is observational in design.
Blinding	Blinding was not possible for participants as they were aware of their vaccination status and this was not a randomized controlled trial.

Reporting for specific materials, systems and methods

We require information from authors about some types of materials, experimental systems and methods used in many studies. Here, indicate whether each material, system or method listed is relevant to your study. If you are not sure if a list item applies to your research, read the appropriate section before selecting a response.

Materials & experimental systems

n/a	Involved in the study
☒	☐ Antibodies
☒	☐ Eukaryotic cell lines
☒	☐ Palaeontology and archaeology
☒	☐ Animals and other organisms
☐	☒ Clinical data
☒	☐ Dual use research of concern
☒	☐ Plants

Methods

n/a	Involved in the study
☒	☐ ChIP-seq
☒	☐ Flow cytometry
☒	☐ MRI-based neuroimaging

Clinical data

Policy information about clinical studies

All manuscripts should comply with the ICMJE guidelines for publication of clinical research and a completed CONSORT checklist must be included with all submissions.

Clinical trial registration

Not applicable.

Study protocol

Not available, as this was a purely observational study.

Data collection

The study participants were recruited from a multicenter healthcare worker cohort from nine healthcare networks in Northern and Eastern Switzerland. Healthcare workers were prospectively included in this cohort and followed since August 2020. All hospital employees aged 16 years and older, with and without direct patient contact, were eligible for inclusion in the cohort upon providing informed consent. In October 2023, all new and ongoing participants in the cohort provided or updated their baseline data. This October 2023 cohort was then used as the study population, with the above mentioned exclusion criteria applied. Weekly follow-up questionnaires were distributed from November 1st, 2023 to April 30th, 2024 in which participants indicated the presence of any of 22 respiratory, gastrointestinal and general symptoms with an acute onset (defined as new occurrence in the preceding 7 days) during the last 7 days, days of absence from work attributable to the symptoms and documented any vaccination against SARS-CoV-2 or seasonal influenza including details on type of vaccine and any test for these pathogens with the results obtained.

Outcomes

Number of episoeds of Influenza-like illness (ILI) was chosen as the main outcome. ILI was defined in accordance with the CDC and ECDC as occurence of fever (≥38.0°C) or feeling of feverishness AND any of the following respiratory symptoms: cough, sore throat, rhinitis or loss of smell, AND an acute onset ≤7 days before the respective reporting date. Sensitivity analyses were perfored using two different case definitions, one being more lenient (acute onset of fever ≥38.0°C or feeling of feverishness AND any of the symptoms asked), one being more restrictive (acute onset of fever ≥38.0°C or feeling of feverishness AND ≥1 general symptom among fatigue, headache, and malaise AND ≥2 other symptoms). As secondary outcome, the number of workdays lost due to these symptoms was examined.

Plants

Seed stocks

Not applicable.

Novel plant genotypes

Not applicable.

Authentication

Not applicable.
